# In vitro inhibitory activity against HPV of the monoterpenoid zinc tetra-ascorbo-camphorate

**DOI:** 10.1016/j.heliyon.2021.e07232

**Published:** 2021-06-06

**Authors:** Ralph Sydney Mboumba Bouassa, Bernard Gombert, Gabin Mwande-Maguene, Aurèle Mannarini, Laurent Bélec

**Affiliations:** a Ecole Doctorale Régionale d’Infectiologie Tropicale de Franceville, BP: 246, Franceville, Gabon; b Laboratoire de Virologie, Hôpital Européen Georges Pompidou, Assistance Publique-Hôpitaux de Paris, 75015, Paris, France; c Faculté de Médecine Paris Descartes, Université de Paris, Sorbonne Paris Cité, 75006, Paris, France; d MGB Pharma, 30900, Nîmes, France; e Faculté de Sciences, Département de Chimie et Biochimie, Université des Sciences et Techniques de Masuku (USTM), BP: 901, Franceville, Gabon

**Keywords:** Terpenoid, Camphor derivates, L-ascorbic acid conjugate, Zn metal, HPV, HPV-16, Pseudovirus, Inhibition assay

## Abstract

Zinc tetra-ascorbo-camphorate (or drug “C14”) is a synthetic monoterpenoid derivative that has potent anti-HIV-1 activity in vitro. In this study, we evaluated the in vitro antiviral properties of C14 against human papillomavirus (HPV). Inhibition assay of HPV-16-pseudovirus (PsVs) adsorption on COS-7 cells by C14 was used. C14 inhibited HPV-16-PsVs adsorption with IC_50_ ranging between 2.9 and 8.3 μM and therapeutic indexes between >410 to >3,300. Pretreatment of COS-7 cells with C14 before addition of HPV-16-PsV was associated with more potent anti-HPV activity than simultaneous deposition on COS-7 of HPV-16-PsV and C14, suggesting that C14 is more effective in preventing HPV attachment to target cells than post-HPV adsorption viral events. Overall, these in vitro studies suggest that the monoterpenoid zinc tetra-ascorbo-camphorate molecule may be suitable for further clinical evaluations as potential microbicide or therapeutic drug.

## 1 Introduction

Human papillomavirus (HPV) infection is the most common viral sexually transmitted infection worldwide and high risk-HPV (HR-HPV) genotypes, particularly HPV-16 and HPV-18, are responsible for 5.2% of all cancers worldwide and 7.7% of all cancers in developing countries [1, 2]. According to the World Health Organization (WHO), early 500,000 women will die of cervical cancer annually worldwide from 2025, especially in countries with limited resources such as in sub-Saharan Africa where it constitutes not only a diagnostic but also a therapeutic management challenge [3, 4].

The prophylactic vaccination with Gardasil-9® vaccine (Merck & Co. Inc., Kenilworth, NJ, USA) containing virus-like particles from HPV-6 and HPV-11, as well as two α7 (HPV-18 and HPV-45) and five α9 (HPV-16, -31, -33, -52 and -58) high-risk HPV, constitutes one of the main strategies against cervical cancer [5, 6], and is subsidized in underserved and poor countries by Global Alliance for Vaccines and Immunization [7]. Although the benefits of global HPV vaccination program are undeniable [7], prophylactic HPV vaccines do not protect against numerous nonvaccine HPV types associated with several HPV-related diseases and have low uptake due, in part, to high cost and cold chain storage requirements [8]. Thus, it is increasingly recognized that the best strategy to prevent HPV infections should combine prophylactic vaccination in addition to topical antiviral chemoprophylaxis [9, 10]. Indeed, molecules used as topical broad-spectrum microbicides targeting HPVs could be useful in preventing HPV sexual transmission, in addition to the prophylactic vaccination [9].

Natural products constitute a wide source of antiviral agents, *a priori* devoid of cellular toxicity [11]. Terpenoids, which derive from isoprene (C_5_H_8_) with a great diversity of chemical structure, are abundant plant metabolites, several molecules of which have therapeutic, anti-tumor and anti-inflammatory properties [12, 13, 14, 15, 16]. Furthermore, terpenoids have generated a lot of interest due to their antiviral properties in vitro against HIV [15, 16].

We previously demonstrated that the zinc tetra-ascorbo-camphorate molecule (named as “C14” drug), a monoterpenoid synthetic derivative, has a potent anti-HIV-1 *in vitro* activity [17]. The development of anti-HPV molecules would overcome the limitations of the current HPV vaccines [18]. This prompted us to evaluate the inhibitory activity of the C14 molecule against HPV. Herein, we have used pseudovirus (PsVs) of HPV-16, the main oncogenic high-risk HPV worldwide [1], to *in vitro* assess the inhibitory activity of C14 against HPV-16-PsVs adsorption on COS-7 cells.

## 2 Material and methods

### 2.1 Zinc tetra-ascorbo-camphorate derivative

**Figure 1:**
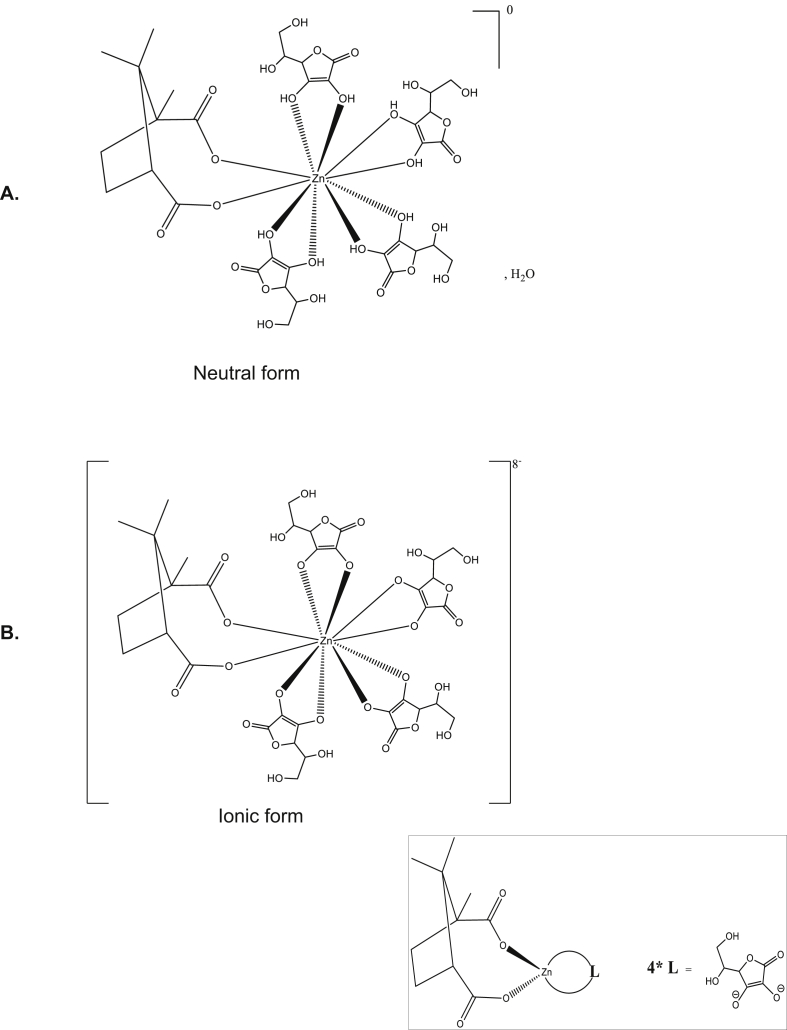
Cram's structure of the neutral (A) and ionic (B) forms of zinc tetra-ascorbo-camphorate 4(C6H6O6)Zn(C10H14O4). The C14 structure comprises a pentacyclic ring including a comphorate terpene [generic formula: (C5H8)n] and 4 L-ascorbic acids stably associated with an unique Zn metal. The box depicts a condensed form of the chemical structure of the C14 molecule solubilized in aqueous or physiologic environments providing aqueous complex negatively charged.

Based on the chemical interactions of L-ascorbic acid with Zn (II) ions in solid state and in aqueous solutions [19], we have provided Cram's structures of the neutral and ionic form of zinc tetra-ascorbo-camphorate in Figures 1A and B, respectively. Zinc tetra-ascorbo-camphorate is a synthetic compound containing a terpenoid system stably associated to 4 ascorbic acids by a Zn atom. The global formula of the zinc tetra-ascorbo-camphorate is as follows: 4(C_6_H_6_O_6_)Zn(C_10_H_14_O_4_). Chemical synthesis of the batch of C14 used in present experiment was previously described in extenso by Saïdi and colleagues [17]. The full detailed synthesis protocol is subjected to a patent and has been verified and approved by an independent laboratory of organic chemistry (Laboratoire de chimie organique, Institut des Sciences Pharmaceutiques et Biologiques, Université Claude Bernard, Université Lyon 1, Lyon, France), attesting to the high purity of the final compound when synthesized according to this procedure (not shown). For this study, the C14 was diluted in ultra-pure Milli-Q® water (Sigma-Aldrich) and the molarity of the stock solution of C14 used was 0.05155 M.

### 2.2 Production of human papillomavirus pseudovirus

The viral lifecycle of HPV is closely linked to the differentiation process of the stratified squamous epithelium of the mucosa [20]. These particularities make HPV very difficult to be cultured in vitro and significantly hinder the assessment of inhibitory activity of a molecule against these viruses. The recent advent of HPV-based pseudoviral particles (PsV) technologies containing a reporter gene has promoted the development of neutralization assay allowing to assess the *in vitro* effect of antiviral candidate or neutralizing antibodies on the early stage of the HPV infection [20]. These viral pseudoparticles are able to bind and penetrate some cell lines and promote the transcription of reporter gene coding for a reporter protein such as green fluorescent protein (GFP) or luciferase [20]. These reporter proteins will react with their specific substrate and only cells inside which the PsV has penetrate will express the reporter protein. Therefore, this method helps to assess the neutralizing activity of compounds against HPV [20]. In the present study, we have produced PsV for HPV-16 according to the recommendations stated by the papillomavirus vectors production protocol (https://ccrod.cancer.gov/confluence/display/LCOTF/PseudovirusProduction) edited by the Laboratory of Cellular Oncology of the Center for Cancer Research (National cancer institute, National Institute of Health, Rockville Pike, Bethesda, Maryland, USA), with minor adaptations, as we fully detailed previously elsewhere [6]. Briefly, the plasmid vector p16sheLL incorporating both L1 and L2 genes for HPV-16 was used to produce HPV-16-PsVs, as previously described [9, 21, 22, 23, 24, 25]. The final PsVs also incorporated the reporter plasmid pGL4.10 [luc2] (Promega, Madison, Wisconsin, USA), encoding the luciferase protein [22]. The plasmid p16sheLL was gently gifted by John Schiller and is available in the Addgene plasmid repository site (Plasmid reference number: # 37320, http://www.addgene.org/). COS-7 cells were used to assess the uptake and to titrate the HPV-16-PsVs produced. Briefly, HPV-16-PsVs incorporate a reporter plasmid pGL4.10 [luc2] encoding the luciferase protein. After the infection of COS-7 with this HPV-16-PsVs, cells are washed to remove the unbound PsVs and incubated with the luciferin, the specific substrate of the luciferase. The luminescence generated by the enzymatic activity of the luciferase carried by HPV-16-PsVs on the luciferin substrate is measured by a luminometer. Therefore, adsorption of HPV-16-PSVs by COS-7 cells is associated with luminescence, while lacks of luminescence indicates the absence of cellular uptake. Dilution of PsVs that yielded at least 80% of the positive control luminescence signal on the luminometer (Luminoskan ascent, Thermo Fisher Scientific, SA, USA), after adding 50 μL of luciferin substrate and after adjusted with background luminescence, were selected as suitable and therefore selected as the working dilution for neutralization experiments.

### 2.3 HPV L1 & L2-based pseudovirus inhibition assay

Serial dilutions of the stock solution of C14 diluted to tenth covering an appropriate concentration range (from 0.0001 to 5,000 μg/mL at working concentrations of 5,000, 500, 50, 5, 0,5, 0,05, 0,005, 0,0005 and 0,0001 μg/mL) were subjected to HPV-16-PsVs-based papillomavirus inhibition assay. Briefly, COS-7 cells were pre-plated (10^4^ cells/well) in Dulbecco's Modified Eagle Medium (DMEM, Thermo Fisher Scientific), supplemented with 10% Fetal Bovine Serum (FBS, Dominique DUTSCHER SAS, France), 100 IU/ml penicillin and 100 μg/ml streptomycin (Gibco, USA) and 50 μg/mL of Fungizone™ Amphotericin B (Thermo Fisher Scientific) in 96-well plates and incubated 24 h in 5% CO_2_ at 37 °C. In the first experimentation, COS-7 cells were preincubated during 3 h in set of C14 dilutions (C14 diluted in non-supplemented DMEM). After the preincubation time, the dilutions of C14 were removed and replaced by HPV-16-PsVs diluted in non-supplemented DMEM. In the second experimentation, the C14 stock solution and HPV-16-PsVs stock were mixed, in the same tube, with non-supplemented DMEM, until reaching the desire concentration for C14 and HPV-16-PsVs into the inoculum (100 μL). Infected cells were grown overnight at 37 °C and then fed with 100 μL of supplemented DMEM. After an additional 24 h of growth at 37 °C, the medium was removed, cells were washed with Dulbecco's phosphate-buffered saline (DPBS) (Thermo Fisher Scientific) and 100 μL of Pierce™ Firefly Luciferase One-Step Glow Assay solution (Thermo Fisher Scientific) was added to each and plate was incubated in the dark, for 15 min. Cells lysates were harvested and transferred to a LumiNunc™ 96-well white microplates and the luciferase enzyme activity was measured as described above. A well containing only the pGL4.10 [luc2] (1 μg) served as negative control (Full luminescent signal) and a background luminescence control well was constituted by only COS-7 cells (no PsV or reporter plasmid). The inhibition of the luminescence signal over 80% was considered as effective inhibition of the PsV transduction of the COS-7 cells. A pool of sera from individuals vaccinated by the Gardasil-9® vaccine (Merck & Co. Inc.) constituted the positive control for the HPV-16-PsVs-based inhibition assay (luminescence signal inhibition of more 80%). All experiments were performed in triplicate.

### 2.4 Cytotoxicity assay

The cytotoxicity of the C14 derivative against COS-7 cells was assessed using MTT assay (Sigma-Aldrich), as previously described [26]. The percentage survival was obtained using the following formula: Survival (%) = live cell number (test)/live cell number (control) x 100. The cytotoxic concentration 50 (CC_50_) corresponding to the C14 concentration (μM) that causes 50% cytotoxicity on COS-7 cells and inhibitory concentration 50 (IC_50_), corresponding to the C14 concentration (μM) that induces 50% inhibition of HPV-16-PsVs activity in luciferase assay in COS-7 cells, were calculated using a dose–response–inhibition analysis on GraphPad Prism v5.04 software (GraphPad Software, San Diego, CA, USA). Then the therapeutic indexes (TI = CC_50_/IC_50_) were calculated.

## 3 Results

### 3.1 High concentrations of C14 are not toxic *in vitro*

**Figure 2:**
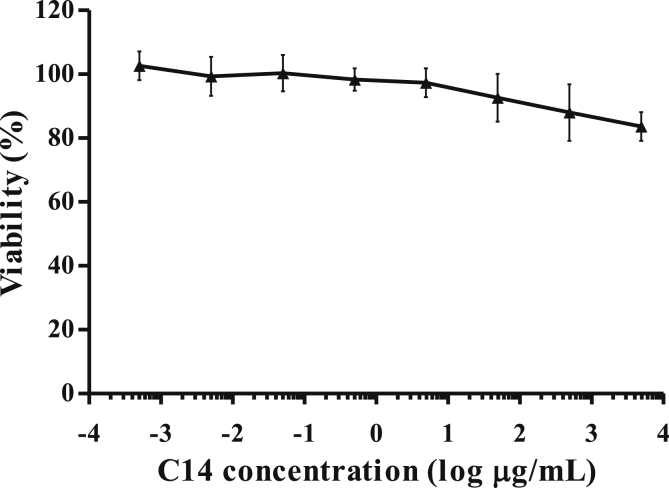
Evaluation of C14 toxicity on COS-7 cells. COS-7 cells were cultured with increased concentrations of C14 for 24 h. After washing, culture viability was determined by using the MTT-cytotoxicity assay according to the manufacturer's instructions. The values given are the mean viability ±1 standard deviation of COS-7 cells, expressed in percentage. Means ± SD are representative of 3 independent experiments.

Therefore, the intrinsic toxicity of C14 derivate concentrations up to 5,000 μg/mL was evaluated by using a colorimetric cell viability assay. COS-7 cells were exposed to serial five-fold dilutions of C14 stock solution for 24 h. The viability index corresponding to the ratio of viable cells after the treatment on the fraction of viable mock-exposed cells was calculated. Cells treated by a solution of PBS-azide 0.1% were used as a positive control for toxicity (data not shown). The viability index corresponding to the ratio of viable cells after the treatment on the fraction of viable mock-exposed cells, was calculated. As shown in Figure 2, C14 demonstrated viability indexes from 0.8 to 1.1 at all concentrations tested, which indicated that it was largely non-toxic. Estimated CC_50_ was >0.00515 M of C14.

### 3.2 HPV-16-PsVs adsorption inhibition by C14 in COS-7 cells

**Figure 3:**
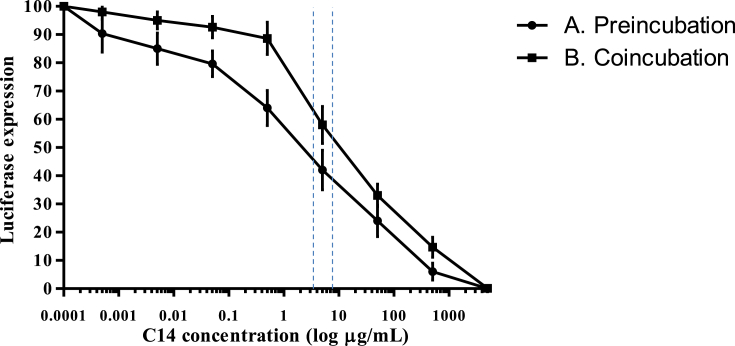
Evaluation of C14 inhibitory activity on HPV-16-PsVs adsorption on COS-7: (A) HPV-16-PsVs was added in culture medium after 3 h preincubation with serial C14 dilutions; (B) HPV-16-PsVs and C14 dilutions were added simultaneously. Pooled sera from individuals having received three doses of Gardasil-9® vaccine (Merck & Co. Inc.) used as positive control showed luminescence signal inhibition above 80% (not shown). The anti-HPV-16-PsVs IC_50_ values (shown as a vertical dotted line) were estimated using the luciferase inhibitory assay in COS-7 cells by the GraphPad Prism v5.04 software (GraphPad Software). Means ± SD are representative of 3 independent experiments. Therapeutic indexes (TI = CC_50_/IC_50_) were between >410 to >3,330.

Figure 3 depicts the inhibition curve of HPV-16-PsVs adsorption by serial dilutions of C14 added at the surface of COS-7 cells during 3 h before adding the PsVs. Estimated IC_50_ was 2.8 μg/mL (2.9 μM) of C14 (95% confidence interval [CI]: [1.5–5.2]), and TIs ranged from >960 to >3,330.

When PsVs was added simultaneously with serial dilutions of C14, the inhibition curve was displaced on the right, with estimated IC_50_ reaching 8.1 μg/mL (8.3 μM) of C14 (95% CI: [6.1–12.1]), and TIs ranged from >410 to >820.

## 4 Discussion

We herein evaluated the in vitro inhibitory activity of the monoterpenoid C14 molecule against the major oncogenic high-risk HPV-16, using an inhibition assay of HPV-16-PsV adsorption on COS-7 cells. C14 exhibited potent inhibition of HPV-16-PsVs adsorption on COS-7 cells, with IC_50_ ranging between 2.9 and 8.3 μM and TIs between >410 to >3,300. Pre-treatment of COS-7 by C14 before adding HPV-16-PsVs was associated with more potent anti-HPV activity than simultaneous deposition on COS-7 of HPV-16-PsVs and C14, suggesting that C14 would act in early stages of the infection by preventing the attachment of HPV on binding sites in target cells, rather than acting directly on the viral post adsorption events. Our observations complement and extent the previous demonstration of *in vitro* efficacy of C14 against against both R5-and X4- HIV-1 [17]. Taken together, the monoterpenoid zinc tetra-ascorbo-camphorate molecule may be suitable for further testing as a broad-spectrum drug candidate to prevent as microbicide male-to-female heterosexual acquisition of HIV-1 or HPV, as well as to be used in the cure of HPV-associated lesions.

The zinc tetra-ascorbo-camphorate molecule belongs to the heterogenous family of terpenoids, which is abundant, diverse in nature, extracted from various plants or obtained by chemical synthesis, and which comprises several characterized therapeutic agents having a large array of pharmacological properties such as anticancer, analgesic, anti-inflammatory, immunomodulatory, and antiviral activities [11, 15, 16]. Antiviral activities of terpenoids have been reported for a wide variety of RNA and DNA viruses, including HIV [11, 16, 17, 27, 28], influenza virus A/WSN/33 (H1N1) [29], or other influenza viruses [15], hepatitis A virus [15, 30], hepatitis B virus [12, 15, 31, 32], hepatitis C virus [15, 33], vesicular stomatitis virus [15], human enterovirus 71 [15], orthopoxviruses [34] pathogenic flaviviruses [35] including dengue virus [36], *Herpesviridae* family viruses [varicella-zoster virus, herpes simplex virus type (HSV) type 1 (HSV-1), Epstein-Barr virus, cytomegalovirus] [15, 31, 33, 37, 38] and emerging pathogenic SARS-coronaviruses [15, 39].

Our observations report, to our knowledge for the first time, on the antiviral activity of a terpenoid against HPV. This may simply be due to the technical difficulties of *in vitro* HPV inhibition tests. Indeed, at this time, HPVs cannot be cultured *in vitro,* nor replicate in a conventional monolayer cell cultures [40, 41]. However, the production of papillomavirus-based gene transfer vectors, known as of HPV L1&L2 based-PsVs, using conventional monolayer cell lines allows the assessment of the initial phases (entry and assembly) of the HPV replication cycle [41].

The mechanism of action of C14 against HPV-16 is yet unknown. It was previously demonstrated that C14 harbors potent HIV-1 entry inhibition activity and/or targets pre-integrative step of viral cycle, indicating that C14 inhibits the initial stages of the HIV replication cycle [17]. In our hands, C14 prevented more efficiently HPV attachment on target cells than HPV post-adsorption events, also suggesting that the inhibitory action of C14 against HPV-16 depends more on the initial stages of viral attachment and penetration into its target cell rather than on later stages of the replication cycle. While further work is needed to determine precisely the molecular mechanism of anti-HPV activity of C14, it is possible to make some assumptions. In the zinc tetra-ascorbo-camphorate molecule, the combination of the monoterpenoid with 4 L-ascorbic acids stabilized by a Zn metal was used to create a new biological terpenoid. Similar strategy of conjugation to create new bioactive terpenoids was previously reported for pentacyclic triterpenoid conjugated with L-ascorbic acid causing increased potent antiviral activity against influenza virus, likely because disruption of the interaction of influenza hemagglutinin (HA) with the sialic acid receptor and thus of the attachment of viruses to host cells secondary to structurally stoechiometric effect of the novel conjugated multi terpenoids complex [42, 43, 44]. Similarly, the conjugation of the monoterpenoid ascorbate of the C14 complex with 4 L-ascorbic acid molecules could have resulted in a spatial configuration capable to alter the first step of host cell infection. In addition, the camphor, an abundant monoterpenoid with a bicyclic framework structure, and camphor derivatives, possess broad antiviral activity [45, 46, 47]. Interestingly, camphor derivatives have demonstrated antiviral activity in vitro against orthopoxviruses belonging to the *Poxviridae* family [34], which is close to the *Papoviridae* family to which HPV belongs. Otherwise, L-ascorbic acid is well known to have anti-inflammatory properties and has demonstrated numerous effects on the immune system [48], and may have indirect anti-viral properties [49, 50]. Furthermore, the four ascorbic acid groups with high polarity [51] likely allow the C14 molecule to be solubilized in aqueous or physiologic environments, providing Zn^2+^-camphorate or hydrated species of zinc-camphorate, which are highly reactive with proteins, as previously described [51, 52]. In this study inhibition assay, aqueous derivative of C14 molecule could interact with the L1 and L2 proteins of HPV-16-PsVs and influence their ability to bind to cells. Such hypothesis could support our findings showing that the pre-treatment of COS-7 before adding HPV-16-PsVs was associated with more potent anti-HPV activity than simultaneous deposition on COS-7 of HPV-16-PsVs and C14.

The risk of HPV infection can be reduced by complementary or even synergistic strategies combining the use of microbicidal molecules and prophylactic vaccines for primary prevention, and early detection and treatment of high risk-HPV-associated lesions [18]. We previously reported the anti-HIV-1 activity of zinc tetra-ascorbo-camphorate molecules on both R5-and X4- HIV-1 infection of primary mucosal target cells and on HIV-1 transfer from dendritic cells to T cells [17]. We herein demonstrated a significant reduction in HPV-16-PsVs infection associated with high TIs when applying C14 3 h before HPV-16-PsV challenge, with IC50 relatively closed to that previously observed against HIV-1 (ranging from 0.02 to 1.3 μM) [17]. This property of high inhibition of the adsorption of HPV-16-PsVs when the C14 is applied in anticipation of exposure to PsVs could be particularly useful in the development of C14 as potential microbicide molecule, to prevent the acquisition of HPV in sexually exposed women. In addition to its antiviral properties against HIV and HPV, C14 has shown inhibitory activity against HSV type 2 (HSV-2) [53]. Thus, the antiviral activity of C14 against the reference strain HSV-2-MS (ATCC® VR-540™) and acyclovir-sensitive clinical HSV-2 and HSV-1 isolates was evaluated using plaque reduction assay on Vero (ATCC: CCL81) and human fibroblast MRC5 cells [53]. Anti-HSV action was further approached by attachment (target cell exposed to the virus in the presence or absence of C14) and penetration (viruses absorbed on pre-chilled cells) assays. C14 inhibited both HSV-2 and HSV-1 replication with IC_50_ ranging between 7.3 and 15.9 μM and selectivity indexes between 170 to 2,500. The simultaneous treatment was more efficient that the post-infection treatment, suggesting that a direct inactivation of viral particles or inhibition of virus replication at the initial phases of the viral replication cycle could be involved. Finally, the cytotoxicity for host primary cells of a non-specific antiviral compound is a major issue. For example, the nonoxynol-9, a non-specific surfactant, which destroys HIV-1 particles *in vitro*, caused lesions in the vaginal epithelium in vivo and increased the probability of being infected with HIV-1 [54]. In addition, the C14 molecule was found to be largely no cytotoxic in vitro at high concentrations against primary mucosal target cells (macrophages, dendritic cells and T cells) and lack of significant inflammation and adverse changes in vivo could be observed in New Zealand White rabbit cervicovaginal tissue integrity after repeated exposure during 10 days to formulations containing C14 [17]. In total, all these observations allow us to hypothesize that the monoterpenoid zinc tetra-ascorbo-camphorate molecule could be used as a potential broad-spectrum microbicide to prevent male-to-female heterosexual acquisition of HIV-1 or HPV. Furthermore, tackling HIV, HSV-2, and HPV with a single strategy may improve anti-HIV efficacy, since HSV-2 and HPV are cofactors of HIV sexual transmission [18], and combining HIV protection with HSV and HPV protection may be beneficial [18]. Such strategies for the use of non-antiretroviral molecules as microbicides with broad-spectrum antiviral activities against HIV, HPV and HSV-2 are already very advanced in clinical trials [10, 18, 55, 56]. This is the case with carrageenan which is highly studied for use as a microbicidal gel that can be applied before sex to prevent sexually transmitted viral infections such as HIV, HSV-2 and HPV [10]. Carrageenan is an extract of red algae which has demonstrated antiviral properties *in vitro* and *in vivo* against HPV (IC_50_: 1–20 ng/mL) [9, 10], HIV (IC_50_: 3 μg/mL) and HSV-2 (IC50: 0.9–3.6 μg/mL) with high TIs [10]. Similar to carrageenan, C14 has also demonstrated antiviral properties *in vitro* against HPV (IC_50_: 2.8 μg/mL), HIV (IC_50_: 1μM) [17], and HSV-2 (IC_50_: 7.3–15.9 μM) [53] with high TI. Finally, the C14 molecule could also potentially be used in the treatment of diseases associated with HPV and HSV-2.

Our study obviously has many limitations. Concerning HPVs, we only evaluated the adsorption inhibition of HPV-16-PsVs on COS-7 cells, and these first observations should be verified with other oncogenic HPV genotypes. The molecular action mechanism of C14 against HPV-16 remains unknown. Further analytical works such as liquid chromatography coupled with mass spectrometry (LC-MS) could be helpful to fully understand the molecular interactions of C14 with HPV-16 viral particle and what happens to C14 after having interacted with the viral particle. In addition, the antiviral activity of monoterpene unit and ascorbic acid, which are the component units of C14, should be examined. Furthermore, our study clearly lacks the demonstration in an animal model that the antiviral activity of C14 molecule *in vitro* is also observed *in vivo*, as for example using the HPV-PsVs vaginal challenge in mice [10, 24, 56].

## 5 Conclusion

Together, our results demonstrate that the C14 is a promising microbicide molecule that should be advanced for further in vivo evaluation and clinical testing.

## Declarations

### Author contribution statement

Ralph Sydney Mboumba Bouassa: Conceived and designed the experiments; Performed the experiments; Analyzed and interpreted the data; Wrote the paper.

Bernard Gombert; Gabin Mwande-Maguene; Aurèle Mannarini: Conceived and designed the experiments; Performed the experiments.

Laurent Bélec: Conceived and designed the experiments; Performed the experiments; Analyzed and interpreted the data; Wrote the paper.

### Funding statement

This research did not receive any specific grant from funding agencies in the public, commercial, or not-for-profit sectors.

### Data availability statement

Data will be made available on request.

### Declaration of interests statement

The authors declare the following conflict of interests: Ralph Sydney Mboumba Bouassa, Gabin Mwande-Maguene and Laurent Bélec report no conflicts of interest. Bernard Gombert, the chief executive officer of MGB Pharma, Nîmes, France, gave the C14 molecule for the study. Aurèle Mannarini is chemist adviser for MGB Pharma, and discussed the interpretation of the results. Bernard Gombert and Aurèle Mannarini did not play a role in the study design, data collection and analysis, as well as decision to publish.

### Additional information

No additional information is available for this paper.
